# Exploring the role of sphingolipid-related genes in clinical outcomes of breast cancer

**DOI:** 10.3389/fimmu.2023.1116839

**Published:** 2023-02-13

**Authors:** Shengbin Pei, Pengpeng Zhang, Lili Yang, Yakun Kang, Huilin Chen, Shuhan Zhao, Yuhan Dai, Mingjie Zheng, Yiqin Xia, Hui Xie

**Affiliations:** ^1^ Department of Breast Surgery, The First Affiliated Hospital of Nanjing Medical University, Nanjing, China; ^2^ Department of Thoracic Surgery, The First Affiliated Hospital of Nanjing Medical University, Nanjing, China

**Keywords:** breast cancer, sphingolipid, PGK1, single-cell sequencing, immunotherapy

## Abstract

**Background:**

Despite tremendous advances in cancer research, breast cancer (BC) remains a major health concern and is the most common cancer affecting women worldwide. Breast cancer is a highly heterogeneous cancer with potentially aggressive and complex biology, and precision treatment for specific subtypes may improve survival in breast cancer patients. Sphingolipids are important components of lipids that play a key role in the growth and death of tumor cells and are increasingly the subject of new anti-cancer therapies. Key enzymes and intermediates of sphingolipid metabolism (SM) play an important role in regulating tumor cells and further influencing clinical prognosis.

**Methods:**

We downloaded BC data from the TCGA database and GEO database, on which we performed in depth single-cell sequencing analysis (scRNA-seq), weighted co-expression network analysis, and transcriptome differential expression analysis. Then seven sphingolipid-related genes (SRGs) were identified using Cox regression, least absolute shrinkage, and selection operator (Lasso) regression analysis to construct a prognostic model for BC patients. Finally, the expression and function of the key gene PGK1 in the model were verified by *in vitro* experiments.

**Results:**

This prognostic model allows for the classification of BC patients into high-risk and low-risk groups, with a statistically significant difference in survival time between the two groups. The model is also able to show high prediction accuracy in both internal and external validation sets. After further analysis of the immune microenvironment and immunotherapy, it was found that this risk grouping could be used as a guide for the immunotherapy of BC. The proliferation, migration, and invasive ability of MDA-MB-231 and MCF-7 cell lines were dramatically reduced after knocking down the key gene PGK1 in the model through cellular experiments.

**Conclusion:**

This study suggests that prognostic features based on genes related to SM are associated with clinical outcomes, tumor progression, and immune alterations in BC patients. Our findings may provide insights for the development of new strategies for early intervention and prognostic prediction in BC.

## 1 Introduction

According to the International Agency for Research on Cancer (LARC), the incidence of BC accounts for 11.7% of all cancers, making it the most prevalent cancer in the world (1). Research on BC has been a hot topic both nationally and internationally, and despite improvements in the early detection and treatment of BC, patients still face serious challenges in terms of poor prognosis (2, 3). To help in BC diagnosis and therapy, it is worthwhile to investigate novel BC prognostic models and discover new biomarkers.

Metabolomics has become widely acknowledged as having a significant impact on the onset and progression of BC throughout the past ten decades (4–8). Sphingolipids are an important component of lipids and SM has become a hot research topic. Sphingolipid metabolism is closely involved in the regulation of apoptosis and proliferation, providing a basis for physiological and pathological studies of various diseases (9–12). Sphingolipid metabolomic is an essential part of cell signaling and is pivotal in regulating the dynamic balance of cell proliferation, differentiation, and apoptosis (13). Ceramide (Cer) and sphingosine (SPH) regulate cell death, senescence, and cell cycle arrest, and sphingosine-1-phosphate (S1P) promotes cell proliferation and possesses anti-apoptotic properties (14, 15). Ceramide and S1P are also important signaling molecules for a variety of basic cellular physiological and biochemical responses such as inflammation, vascular endothelial barriers, immune cell transport, stress response, apoptosis, and autophagy, and are involved in regulating vital activities such as angiogenesis and smooth muscle contraction and diastole (16, 17). Recently, abnormal levels of sphingolipid molecules have been detected in the serum of BC patients, suggesting a corresponding role of sphingolipid molecules in the development of BC (18, 19). However, the association between SM and breast cancer biology and clinical outcomes is not fully understood. Therefore, understanding the specific mechanisms and targeting interventions are crucial for BC diagnosis and treatment, and the use of SRGs to predict treatment efficacy and clinical prognosis deserves further study.

Single-cell sequencing analysis is a new sequencing method, which has attracted many researchers because of its accuracy. Single-cell sequencing gives us a way to precisely assess gene expression at the cellular level (20). Bioinformatics analysis of single cells and transcriptome sequencing for cancer immune microenvironment (TME) analysis and survival analysis are important analytics as they provide new biomarkers for precision cancer diagnosis and treatment. Risk profiles are widely used to predict prognostic outcomes in various types of cancer, and risk profiles constructed across multiple cancer types have been shown to outperform traditional methods (including pathology and imaging estimates) in predicting the clinical prognosis (21–23). Therefore, it is of clinical importance to explore new prognostic features.

We downloaded BC public data for this study from the TCGA and GEO databases. Through comprehensive bioinformatics analysis, a new prognostic model was finally constructed using 7 SRGs. Based on risk levels, BC patients were split into two groups: high- and low-risk. Additionally, in BC patients, changes in immune infiltration and immunological checkpoints can be detected using the sphingolipid metabolic profile. Our study might offer a fresh perspective on the investigation of BC diagnosis and care.

## 2 Materials and methods

### 2.1 Transcriptome data acquired and processing

Breast cancer RNA expression profiles, gene mutation, and corresponding clinical data were retrieved from the TCGA database (n=1095) and divided into a training group and validation group by 7:3, in which the training group was used to construct the model, and the validation group was used to check the stability and accuracy of the model. Simultaneously, the GEO expression profiles of GSE20685 were downloaded for use as an external independent validation cohort. All data were in TPM format and log2 was transformed for subsequent analysis. Adjustments for the batch effect between TCGA-BC and GSE20685 were made with the “sva” package.

### 2.2 scRNA-seq data acquired and processing

From the GEO database, the single-cell data set GSE161529 of BC was retrieved. There are ten samples in all in the dataset. We performed the quality control of scRNA-seq data by “seurat” and “singleR” R packages. We kept cells with less than 10% mitochondrial genes, cells with more than 200 genes overall, and genes whose expression spanned from 200 to 7000 and were expressed in at least three cells to keep high-quality scRNA-seq data. A total of 50,917 eligible cells were selected for further exploration. The remaining cells were further scaled and normalised using a linear regression model with the “Log-normalisation” technique. After data normalization, the top 3,000 hypervariable genes were distinguished according to the “FindVariableFeatures” function. As these data were obtained from several samples, we utilised the “FindlntegrationAnchors” function of the canonical correlation analysis (CCA) method to eliminate the batch effects disrupting downstream analysis. Subsequently, we used the “IntegrateData” and “ScaleData” functions to adequately integrate and scale the data, respectively.

Anchor points were identified by principal component analysis (PCA) dimensionality reduction. The t-distributed stochastic neighbour embedding (t-SNE) approach was used to examine the top 20 PCs to find meaningful clusters. Cell cycle heterogeneity along the clusters was evaluated based on the cell cycle markers embedded in the “seurat” package.

### 2.3 The acquisition of sphingolipid-related genes

The GeneCards database served as a source for sphingolipid-related genes, and a total of 110 SRGs with a relevance score greater than 1.0 were selected for subsequent investigation.

### 2.4 AUCell

scRNA-seq data were used to obtain the most relevant genes affecting sphingolipid metabolic (SM) activity. The “AUCell R” package, which determines the active status of gene sets in scRNA-seq data, was employed to assign sphingolipid activity scores to each cell lineage. The percentage of highly expressed gene sets in each cell was estimated using the gene expression rankings of each cell based on the area under the curve (AUC) value of the selected SRGs. AUC values were larger for cells that expressed more genes. Cells actively involved in sphingolipid gene sets were determined using the “AUCell explore Thresholds” function. The cells were then divided into high- and low-sphingolipid-AUC groups based on the median AUC score and visualizes using the “ggplot2 R” tool.

### 2.5 Single sample gene set enrichment analysis

To calculate the precise score of a gene set enriched in a sample, ssGSEA analysis was frequently utilised. In this study, ssGSEA analysis was used to determine the SM scores for each TCGA-BC patient.

### 2.6 Weighted co-expression network analysis

The “WGCNA” package in R implements WGCNA, a systems biology technique for creating the TCGA-BC gene co-expression network. Based on the interconnectivity of each gene set and the relationship between the gene set and the phenotype, WGCNA can be used to find highly covarying gene sets and to identify possible biomarker genes or therapeutic targets. In this work, WGCNA was used to identify the gene modules associated with SM score in BC and to identify the associated genes.

### 2.7 Establishment of a risk signature associated with sphingolipid

First, a univariate Cox analysis was used to extract the sphingolipid-related genes having prognostic value. The prognostic model was then built after the Lasso regression was used to further screen prognostic SRGs. Each BC can therefore be given a risk score using the algorithm in this manner. Based on the median value, patients in the TCGA-BC cohort were divided into high- and low-risk groups. Then, we investigated how the two groups’ prognoses varied from one another and evaluated the model’s precision.

### 2.8 Independence and validity assessment of the prognostic model

To calculate the probabilities of OS at 1, 3, and 5 years, we developed a nomogram combining the risk score, age, gender, pathological stage, and other clinical parameters as independent prognostic factors. In the meantime, survival curves were plotted using the Kaplan-Meier method for prognostic reasons, and log-rank tests were run to assess the statistical significance (24). To assess the nomogram’s accuracy, calibration and ROC curves were created. Using decision curve analysis, we further assessed the net benefit of the nomogram and clinical features alone (DCA). To assess the prognostic significance of risk score clinical features, stratified analysis was used (age, gender, clinical stage, and pathological T stage).

### 2.9 Analysis of the correlation between prognostic models and tumor immunity and immunotherapy

We determined the degree of immune infiltration for BC patients in the TCGA database from the TIMER 2.0 database, which contains the results of seven evaluation methods (25). Heatmaps were created using these data to quantify the relative proportions of immune cell infiltration in the TME. Subsequently, ssGSEA analysis of genes in the prognostic risk assessment model was carried out using the R package GSEABase with immune-related properties (26). The “estimate” R package allows users to determine the relative abundance of stromal cells, immune cells, and tumor cells to then compare these values across different risk categories.

### 2.10 Mutational landscape and drug sensitivity

The “maftools” software was used to generate the gene mutation profiles of BC patients after they were retrieved from the TCGA database. The detailed gene mutation files were merged with the risk score. Additionally, we calculated the half-maximal inhibitory concentrations (IC50) of common chemotherapeutic drugs using the R package “pRRophetic,” which allowed us to assess the relationship between the risk score and drug sensitivity. IC50 values were compared between the two risk groups using Wilcoxon signed-rank tests.

### 2.11 Cell culture and tissue collection

The First Affiliated Hospital of Nanjing Medical University provided the tissue samples, which were stored at -80°C. Twenty tissue pairings, including tumor tissue (T) and precancerous tissue (N), were collected from BC patients undergoing tumor resection between February 2021 and March 2021. Our hospital’s Institutional Ethical Board gave the study its approval (2010-SR-091). The clinical sample information of 20 pairs of patient tissues was presented in **Supplementary Table S1**. The Cell Resource Center of Shanghai Life Sciences Institute provided the HBL-100 human normal breast epithelial cell line and the MDA-MB-231, HCC1806, MCF-7, and BT-474 human BC cell lines. These cells were cultured in DMEM or RPMI-1640 (Gibco BRL, USA). All cells were grown at 37°C with 5% CO2 in 10% fetal bovine serum (FBS) from Gibco BRL in the United States.

### 2.12 Reverse transcription-quantitative PCR

RNA was isolated using the TRIzol reagent (Invitrogen, Carlsbad, California). Using the HiScript RT Mix (Vazyme, Nanjing, China), total RNA (500ng) was further reverse transcribed into cDNA. Quantitative real-time PCR (qRT-PCR) was performed using the SYBR Green Kit (Vazyme, Nanjing, China). The internal controls were GAPDH. Tsingke Biotech (Beijing, China) designed all primers, and detailed primer sequences were presented in **Supplementary Table S2**.

### 2.13 RNA interference

RiboBio created the PGK1-targeting siRNAs and the accompanying negative controls (Si-NC) (Guangzhou, China). To transfect siRNAs, Invitrogen’s Lipofectamine 3000 was utilised. Through RT-qPCR, the transfection effectiveness was validated. **Supplementary Table S2** contains a list of the sequences.

### 2.14 Cell proliferation assay

The Cell Counting Kit-8 (CCK-8; Vazyme, Nanjing, China) was used to detect cell proliferation. We seeded the cells in 96-well plates at 2×10^3^ cells per well. The plate was then incubated with 10 μl CCK-8 labeling reagent (A311-01, Vazyme, Nanjing, China) per well for 2 hours in the dark at 37°C. The absorbance of the cells was measured at 450 nm wavelength with the enzyme-labeled meter (A33978, Thermo, USA) to analyse the viability of the cells. It was detected for 0, 24, 48, 72, 96, and 120 hours.

### 2.15 Colony formation

We transfected 1000 cells and kept them in 6-well plates for approximately 14 days. Two weeks later, we saw the cell clones with the naked eye. Next, the cells were rinsed and fixed for 15 minutes in 4% paraformaldehyde (PFA). Crystal violet (Solarbio, China) staining was performed for 20 minutes, dried at room temperature, and counted per well.

### 2.16 Transwell assay

Transwell experiments included cell migration and invasion experiments. In the upper chamber, treated cells were cultivated in a 200 μl serum-free medium with 2×10^4^ cells per well. To assess the cells’ ability to invade and migrate, the upper portion of the plate was either precoated with Matrigel solution (BD Biosciences, USA) or left untreated. Additionally, a bottom chamber with 600μL of 10% serum medium was available. The cells were fixed with 4% PFA, stained with 0.1% crystal violet (Solarbio, China), and counted under a light microscope.

### 2.17 Animal models

The Committee on the Ethics of Animal Experiments at Nanjing Medical University gave its approval to all animal experiments. For the xenograft model, female BALB/c five-week-old BALB/c mice were used. MDA-MB-231 cells that were stably transfected with PGK1 and control cells were implanted into the left and right groins of the mice independently for tumorigenicity tests. Every five days, the tumor weights and volumes were assessed. The xenograft tumors were separated from their surrounding tissue and weighed 25 days following the injection.

### 2.18 Statistical analysis

Software called GraphPad Prism (version 8.0) was used to analyse experimental data. Three independent experiments recorded the data as mean ± standard deviation (SD). We tested the comparisons among the groups with Student’s t-tests (*P<0.05, **P<0.01, ***P<0.001).

## 3 Results

### 3.1 Single-cell sequencing data analysis

**Figure 1:**
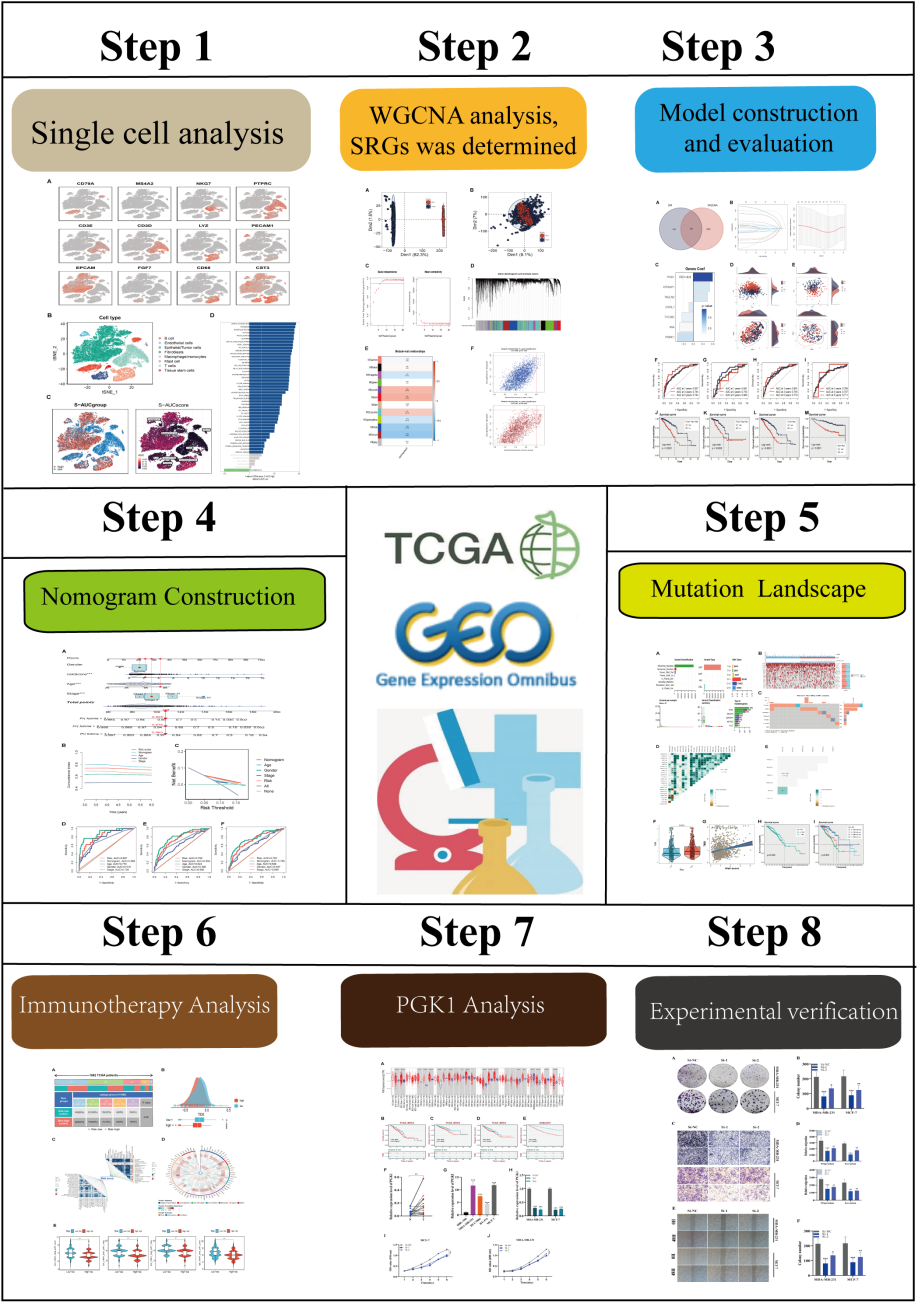
The flowchart of this study.

**Figure 2:**
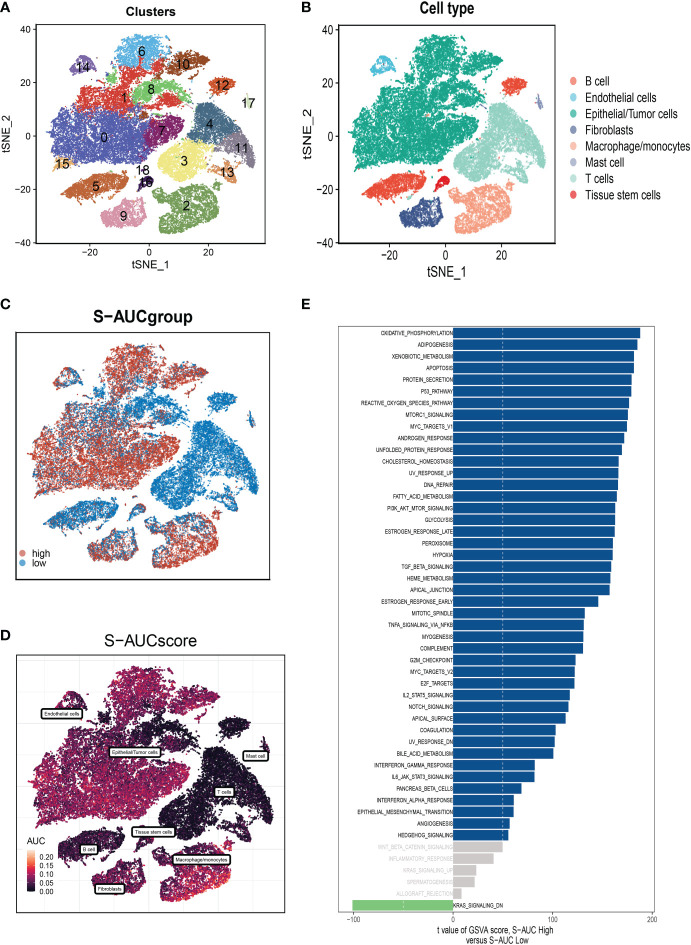
Annotation of cell subsets and identification of differentially expressed genes. **(A)** The results of the dimension reduction cluster analysis are shown in the tSNE diagram. **(B)** Cells were annotated into 8 different types of cells. **(C, D)** All cells were scored according to sphingolipid-associated genes (SRG) and were divided into high and low groups. **(E)** Analysis of differentially expressed genes between high and low groups.

**Figure 1** displayed the study’s flowchart. On the single-cell data set, we conducted quality control. To confirm the validity of the cell samples, as seen in **Supplementary Figure S1A**, we removed some cells and restricted the percentage of mitochondrial genes, ribosomal genes, and red blood cell genes. Sequencing depth and total intracellular sequences exhibit significantly substantial positive associations (R=0.92, **Supplementary Figure S1B**). The PCA reduction plot did not reveal any appreciable variations in cell cycles (**Supplementary Figure S1C**). The study contained 10 samples, where each sample’s cell distribution was largely constant. This suggests that there was no noticeable batch impact on the samples, which might be used for further analysis (**Supplementary Figure S1D**). **Supplementary Figure S1E** displays the expression of the genes that identify the cell type. Subsequently, all cells were classified by the dimensionality reduction algorithms, namely, t-SNE into 19 more detailed clusters (**Figure 2A**). There are eight different types of cells, such as Fibroblasts, Monocytes/macrophages, and tumor cells (**Figure 2B**). The AUCell R package was used to determine each cell’s SM activity to explore the SRGs’ expression features. Higher AUG values were seen in cells that expressed more genes, which were primarily orange-colored B cells and plasma cells (**Figures 2C, D**). All cells were assigned an AUC score for the corresponding SRGs and divided into two groups (high-and low-sphingolipid-AUC groups) by AUC score threshold values. To elucidate the potential biological mechanisms of distinct AUC scores, we performed differential and functional analyses to identify DEGs and pathways related to glycosylation between high-and low-sphingolipid-AUC subgroups. We identified 1,221 genes most likely to influence SM by differential analysis. These terms were mainly related to oxidative phosphorylation, apoptosis, fatty acid metabolism, and the p53 pathway (**Figure 2E**).

### 3.2 Weighted co-expression network analysis

**Figure 3:**
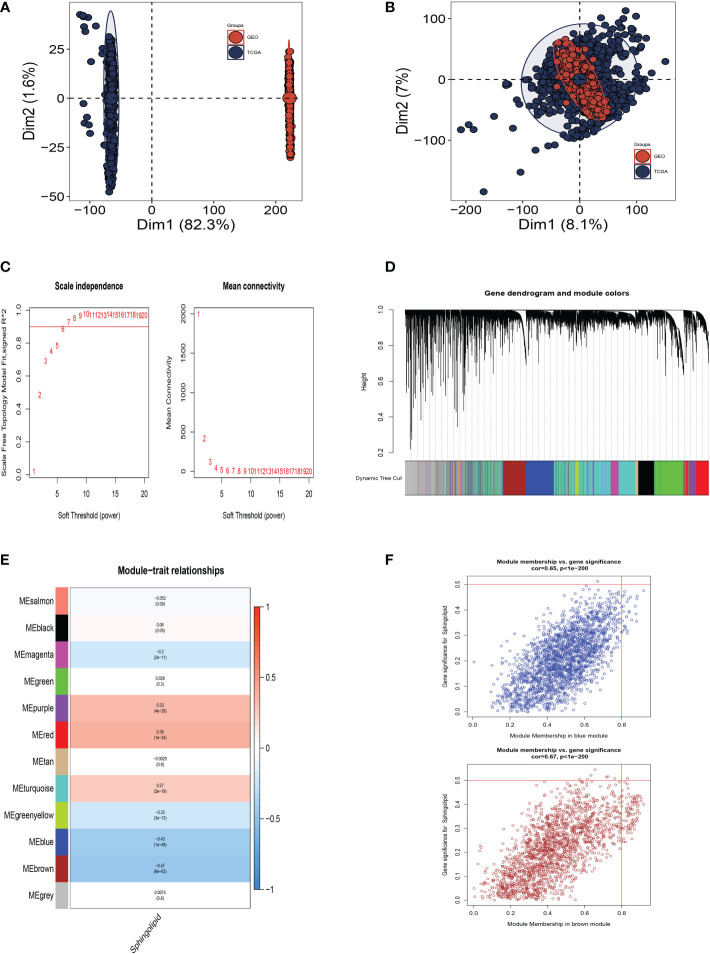
Weighted Co-Expression Network Analysis. **(A)** No significant batch effects were observed in the TCGA cohort and GEO cohorts. **(B)** Removing the batch effect. **(C–F)** Weighted Co-Expression Network Analysis. The blue and brown modules were most associated with sphingolipids, of which 3,787 genes were extracted.

**Figure 3A** shows that TCGA and GEO cohorts independently, with significant batch effect. After removing the batch effect, more accurate results were obtained (**Figure 3B**). WGCNA was used to look for gene sets that were covarying with sphingolipid in more detail. As seen in **Figure 3C**, the data is more consistent with the power-law distribution and the mean connectivity tends to be stable when the soft domain value is 6; making the data suitable for further study. As seen in **Figure 3D**, 12 non-gray modules were generated after merging the modules with a similarity lower than 0.25 and setting the minimum number of modules to 100 and deepSplit to 2. According to **Figures 3E, F**, we discovered that the blue and brown modules, which each contained 3,787 genes, were most closely related to SM (COR = 0.65, *P<*0.001).

### 3.3 Construction and validation of sphingolipid-related prognostic model

**Figure 4:**
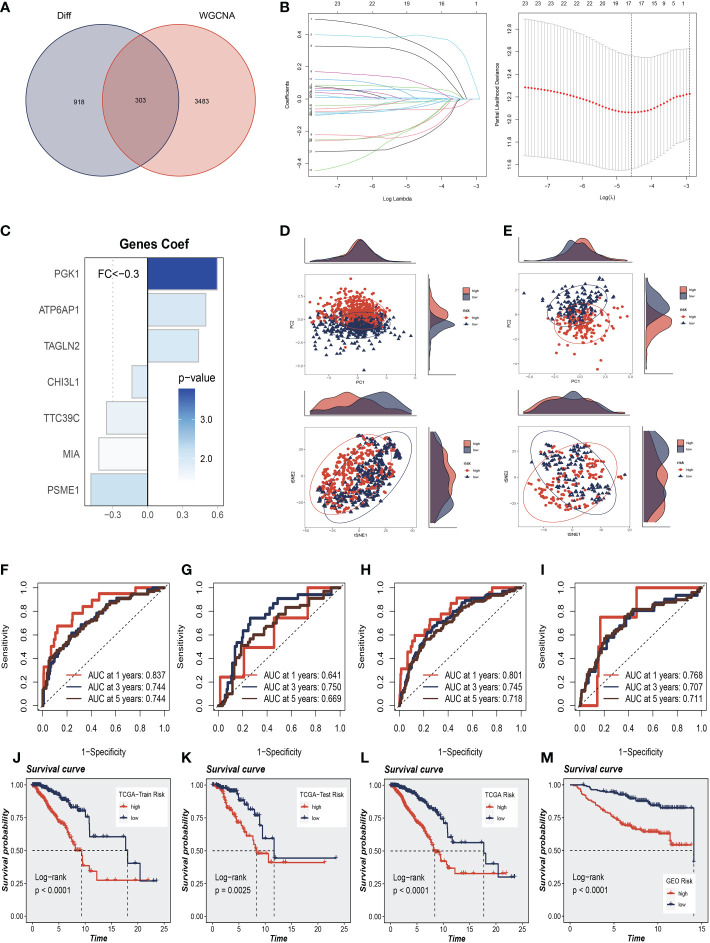
Construction and Validation of Sphingolipid-Related Prognostic Model. **(A)** The intersection of genes obtained in single-cell analysis and bulk-RNA analysis. **(B)** LASSO Cox regression analysis to develop the prognostic model. **(C)** The role of seven model genes. **(D, E)** PCA and t-SNE analysis in the training set and validation set, respectively. The seven model genes did a more accurate job of dividing patients into two groups. **(F–H)** The area under the curve (AUC) values for the TCGA train, test, and full cohort. **(I)** The areas under the curve at 1, 3, and 5 years for the GEO test group. **(J–L)** Survival analysis in the TCGA train, test, and entire cohort (P<0.001). **(M)** Survival analysis in the GSE20685 test cohort.


To further explore how SRGs relate to the prognosis of BC patients, we intersected the most relevant genes affecting sphingolipid metabolic activity obtained in single-cell. Furthermore, Bulk-RNA analysis and 303 genes were used for subsequent analysis (**Figure 4A**). We used the training set in TCGA-BC for model construction, and a total of 63 prognostic genes were obtained by univariate analysis (*P*<0.01). Next, LASSO Cox regression analysis was employed to develop the prognostic model (**Figure 4B**). A total of seven model genes (TAGLN2, CHI3L1, PGK1, ATP6AP1, MIA, PSME1, TTC39C) were finally screened out under optimal regularisation parameters. The prognostic model was calculated as follows:

$$risk\;score=\sum_{n=i}^{k}\left(Coef_{i}Exp\;i\right)$$

Coefi and Expi represented the coefficient and expression of each model gene, respectively, and the risk score for each sample was calculated by the above formula. By using the aforementioned formula, the risk score for each sample was determined. Based on median values, patients were split into high-risk and low-risk groups. Of the seven genes used to construct the model, three were risk factors and four were protective factors (**Figure 4C**). It was discovered that the model could effectively group BC patients in both the training cohort and the test cohort by performing PCA and t-SNE evaluation of the model’s seven genes in the training set and validation set, respectively (**Figures 4D, E**). We performed ROC curve analysis in both the training and test cohort to further investigate the precision of sphingolipid in the assessment of the prognosis of BC patients. The area under the curve (AUC) values for the TCGA train, test, and full cohort were all more than 0.7 (**Figures 4F–H**). We discovered that the areas under the curve at 1, 3, and 5 years for the GEO test group were 0.768, 0.707, and 0.711, respectively (**Figure 4I**). In **Figures 4J–L**, we discovered that the high-risk group had a poor prognosis in the TCGA train, test, and entire cohort (*P*<0.001). Similarly, we saw that patients in the high-risk group in the GSE20685 test cohort had a considerably worse prognosis than those in the high-risk group (*P*<0.001, **Figure 4M**). This shows that the prognostic model related to sphingolipids is highly accurate at predicting patient outcomes in both cohorts.

### 3.4 A nomogram’s construction

**Figure 5:**
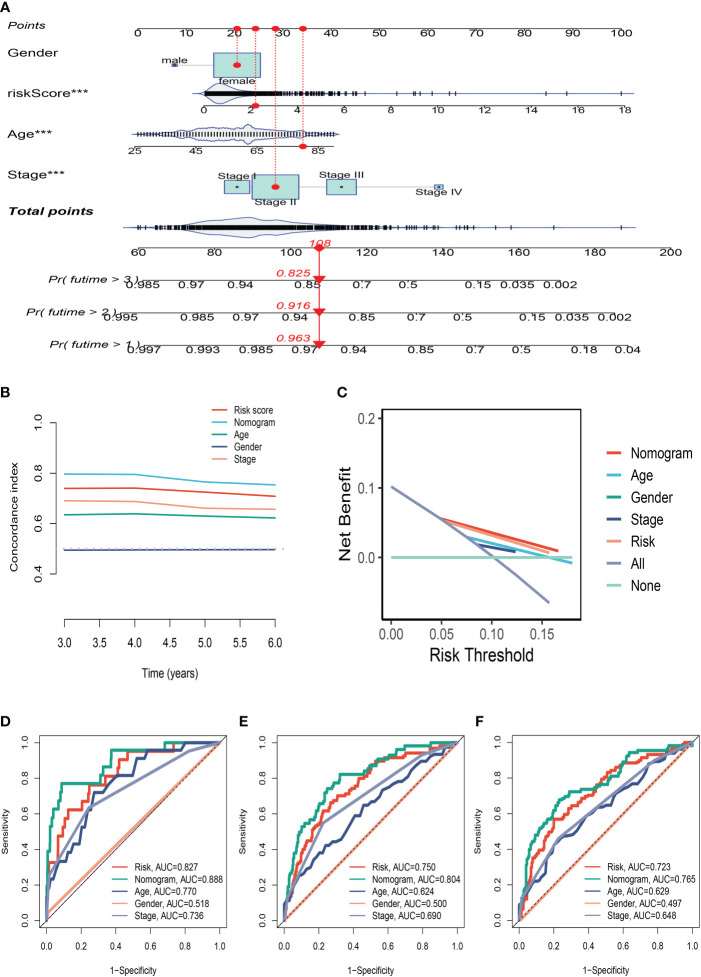
The Construction of a Nomogram. **(A)** Nomogram to assess the risk of BC patients. **(B)** Decision curve. **(C)** Concordance index study. **(D–F)** Prognostic ROC analysis in 1, 3, and 5 years, respectively. ***P<0.001.

Using clinical information and a risk score, a nomogram was created to more accurately quantify the risk of BC patients (**Figure 5A**). The nomogram can help determine patient risk more accurately and direct future treatment decisions. We also carried out the decision curve and concordance index study, which determines the area of each clinical feature and none’s horizontal axis to assess the clinical decision value. Results indicated that this nomogram’s efficacy was superior to that of other clinical indicators, indicating that it is effective in forecasting patients’ prognoses and can serve as a clinical decision-making tool (**Figures 5B, C**). Prognostic ROC analysis was carried out to thoroughly assess the accuracy of this nomogram. According to the findings, the area under the curve (AUC) was 0.888, 0.804, and 0.765 in 1, 3, and 5 years, respectively (**Figures 5D–F**).

### 3.5 Clinicopathological analysis of the prognostic signature in BC

**Figure 6:**
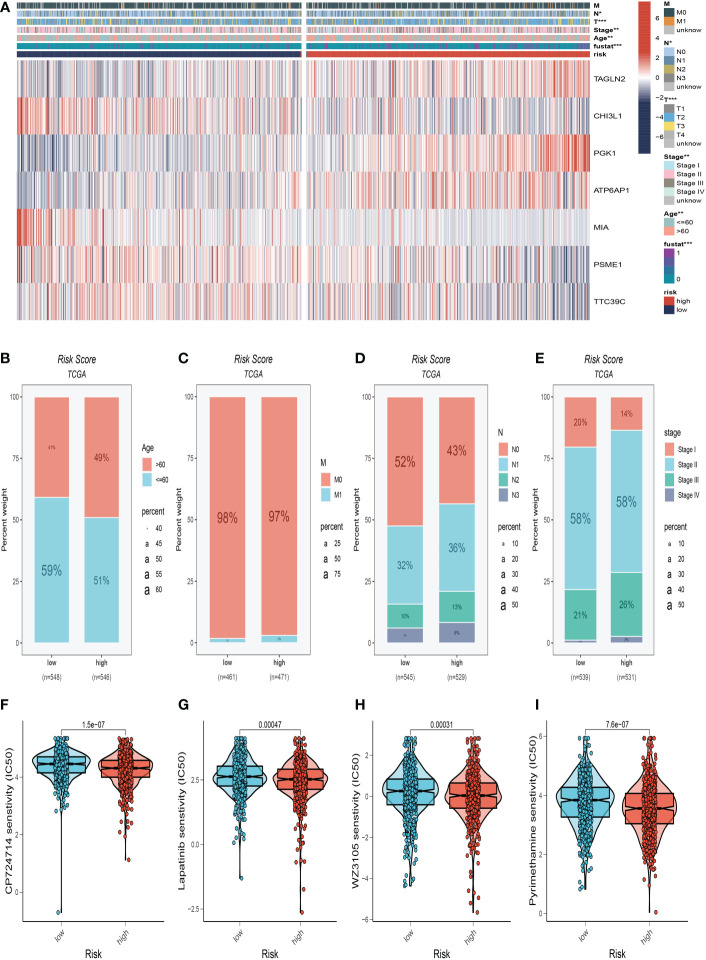
Clinical correlation analysis. **(A)** There were significant differences in N stage, T stage, total stage, and survival between high and low-risk groups. **(B)** The age difference of patients between high and low-risk groups. **(C)** M stage difference of patients between high and low-risk groups. **(D)** N stage difference of patients between high and low-risk groups. **(E)** Total stage difference of patients between high and low-risk groups. **(F–I)** Potential drug screening in high-risk patients. *P<0.05, **P< 0.01, ***P<0.001.

We made a clinical heat map to determine the differences in clinical features between the two risk groups. **Figure 6A** shows noteworthy differences in tumor age, T, and N stage (*P*<0.05) between these two groups. Interestingly, there were older age patients and more advanced N, M stage patients in the high-risk group (**Figures 6B–E**). The differences in drug resistance between the two groups were further discussed and presented in **Figures 6F–I**. We found that CP724714, Lapatinib, WZ3105, and Pyrimethamine may be candidates for treating drugs for the high-risk group. This will provide a reference for choosing the most suitable drugs for clinical practice.

### 3.6 Mutation landscape analysis

**Figure 7:**
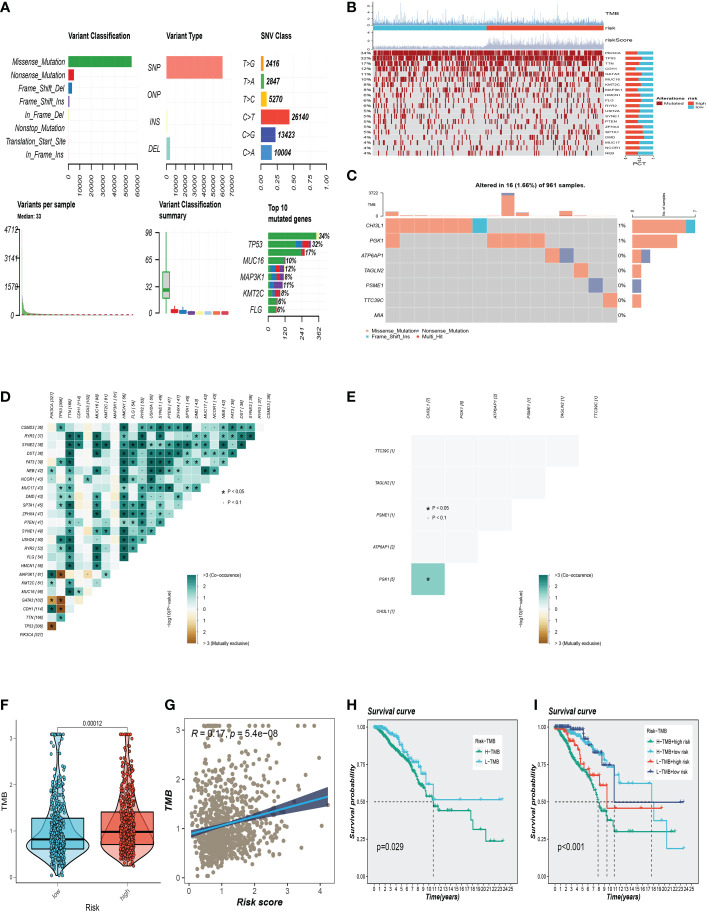
Gene mutation analysis. **(A)** Mutation landscape in the BC samples. **(B)** The representative gene variants in the groups at high and low-risk groups. **(C)** The mutations of 7 model SRGs. **(D)** The mutation symbiosis of the top 25 genes. **(E)** The mutation symbiosis between CHI3L1 and PGK1. **(F)** Differences in tumor mutation burden (TMB) levels between the two risk groups. **(G)** The correlation between TMB and risk score. **(H, I)** Correlation analysis between TMB and prognosis.

The overview of mutations in the BC samples was exhibited in **Figure 7A**, among which the most common mutation type was a missense mutation. The top 3 most frequent mutant genes were TP53, MUC16, and MAP3K1. We also examined representative gene variants in the groups at high and low risk (**Figure 7B**). Genes such as TP53, GATA3, ZFHX4, SPTA1, and DMD had the top five mutation frequencies in the high-risk group. The top five genes with the highest mutation frequencies in the low-risk group were PIK3CA, CDH1, MAP3K1, PTEN, and NEB respectively. **Figure 7C** analysed the mutations of 7 SRGs used to construct the model in 961 BC samples, among which CHI3L1 and PGK1 had mutations. Furthermore, we examined the mutation symbiosis of the top 25 genes and discovered that PIK3CA and NEB, MAP3K1, KMT2C, GATA3, CDH1, and TP53 all shared a mutation symbiosis (*P*<0.05, **Figure 7D**). Furthermore, we found a mutation symbiosis between CHI3L1 and PGK1 (**Figure 7E**). The levels of tumor mutation burden (TMB) between the two risk groups differed significantly, and there was a positive connection between risk ratings and TMB values (**Figures 7F, G**). We investigated the prognostic impact of TMB combined with the two groups on OS. Survival analysis suggested that higher TMB levels were relevant with worse OS (**Figures 7H, I**).

### 3.7 Immune landscape and immunotherapy

**Figure 8:**
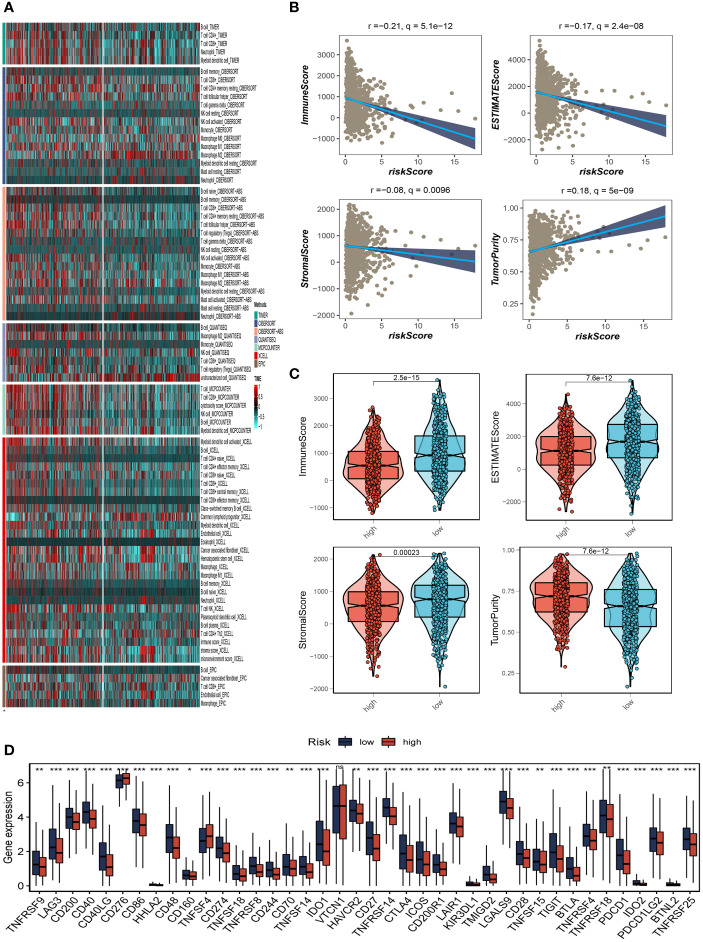
Analysis of immune microenvironment. **(A)** The distribution and association of the 22 tumor-infiltrating immune cells (TICs) in the TCGA-BC cohort. **(B, C)** Correlation analysis of immune score and risk score, ESTIMATE score and risk score, Stromal score and risk score, tumor purity and risk score. **(D)** Differences in the abundance of immune-checkpoint-related genes between high and low-risk groups. *P<0.05, **P< 0.01, ***P<0.001, ns indicates No significance.

We determined the degree of immune cell infiltration in each sample using the CIBERSORT method to better understand the distribution and association of the relative content of 22 tumor-infiltrating immune cells (TICs) in the TCGA-BC cohort. Except for uncharacterised cells, common lymphoid progenitor cells, and M2 macrophages, the low-risk group appeared to have larger levels of immune infiltration than the high-risk group (**Figure 8A**). The low-risk group then had higher stromal scores, immunological scores, and ESTIMATE scores (*P*<0.001), indicating a higher overall immune level and immunogenicity of the TME in that group. We also looked at tumor purity, and the results showed a positive correlation between the two (**Figures 8B, C**). Due to the significance of immune checkpoints for immunotherapy’s success in tumors, we also looked into how immune checkpoint expression varied between the two groups. 37 immune checkpoint genes were significantly upregulated in low-risk patients. A substantial elevation of the immunological checkpoint genes CD276 and TNFSF4 was observed in the high-risk group (**Figure 8D**). Patients with this subtype of the tumor might benefit from targeted therapy against immunological checkpoints that have increased expression.

### 3.8 SRGs risk score predicts treatment response assessment

**Figure 9:**
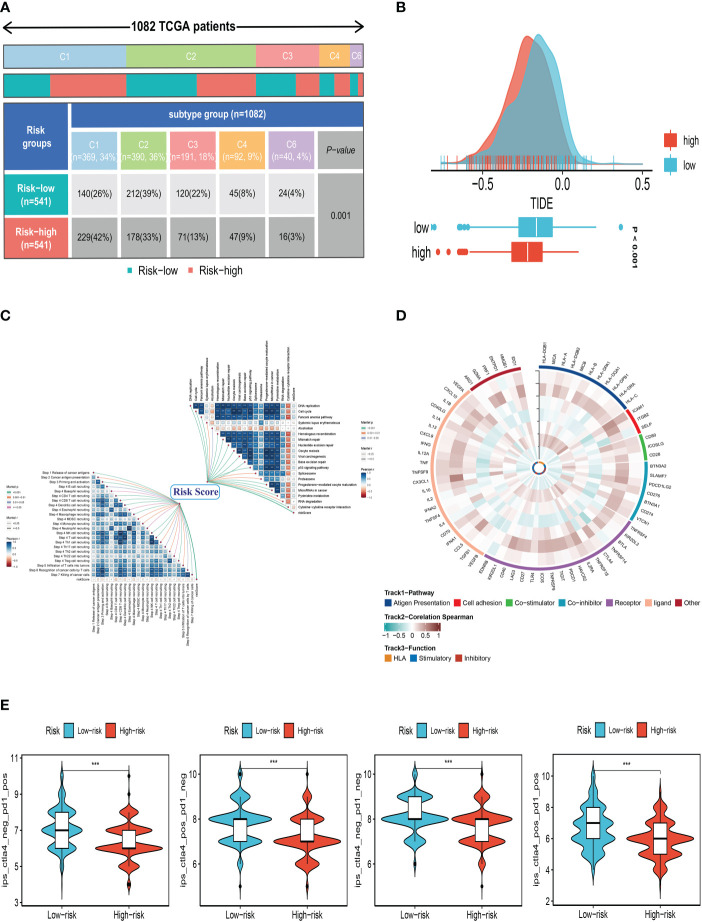
Correlation analysis of treatment response. **(A)** Immune typing of high and low-risk groups. **(B)** The difference in TIDE scores between high and low-risk groups. **(C)** The relationship between SRG risk scores and positive immune checkpoint blockade (ICB) related signals. **(D)** CHI3L1 and ATP6AP1 may affect the prognosis of patients by changing the state of the tumor microenvironment. **(E)** Differences in IPS reactivity between high and low-risk groups. ***P<0.001.

We first compared the immune typing of the high and low-risk groups with the traditional immune typing. We compared the immunological subtype distributions of BC in various risk categories. The findings showed that the immunophenotyping of the various groups varied significantly (**Figure 9A**). Regarding how TMB and immunotherapy interact, to determine if patients with various risk patterns respond to immunotherapy differently, a tumor immune dysfunction and exclusion (TIDE) analysis was performed. According to the findings, the high-risk group responded to immunotherapy better since they had a lower TIDE score (**Figure 9B**). The relationship between SRG risk scores and positive immune checkpoint blockade (ICB) related signals was then further investigated. The findings demonstrated a substantial positive correlation between risk scores and DNA replication, cell cycle, the Fanconi anemia pathway, homologous recombination, mismatch repair, and nucleotide excision repair (**Figure 9C**). It can be seen from **Figure 9C** that there is a significant positive correlation between CHI3L1 and immune-related genes. It can be seen from the previous analysis that the HR of CHI3L1 is 0.87415, thus it can be speculated that CHI3L1 may be a key gene to activate the adaptive immune response in the tumor microenvironment and that ATP6AP1 is significantly negatively correlated with immune genes. From the previous analysis, it can be seen that the HR of ATP6AP1 is 1.64752, so it can be speculated that ATP6AP1 may have an inhibitory effect on the immune response, thus promoting the growth and metastasis of tumors. From the above analysis, we can estimate that CHI3L1 and ATP6AP1 can determine the prognosis of patients by changing the state of TME, which may provide a new idea for clinical treatment (**Figure 9D**). Furthermore, IPS can contribute to screening patients who are susceptible to immunotherapy. In our research, the low-risk subtype has higher IPS and blocker scores than the high-risk subtype, highlighting that low-risk patients may be more susceptible to immune checkpoint inhibitors (ICIs) treatment and derive more significant benefits (**Figure 9E**).

### 3.9 Expression and prognosis of PGK1 in BC samples

**Figure 10:**
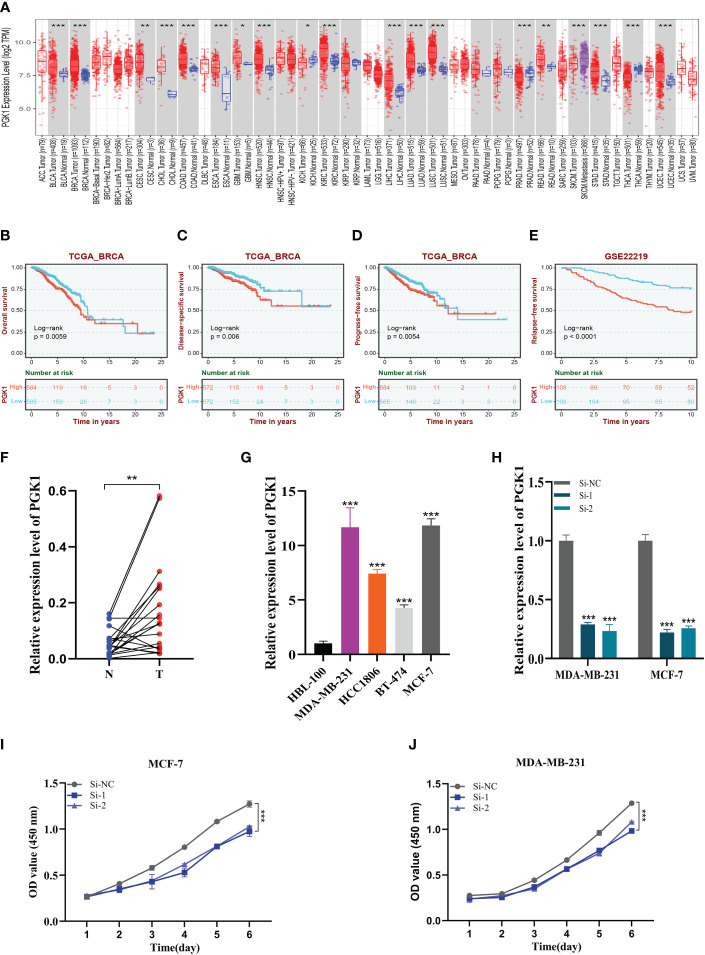
Expression analysis and experimental validation of PGK1. **(A)** Pan-cancer expression profile of PGK1. **(B–D)** The overall survival (OS), disease-specific survival (DSS), and progression-free survival (PFS) analysis of PGK1 in the TCGA cohort. **(E)** The Relapse free survival (RFS) analysis of PGK1 in the GSE22219 cohort. **(F)** PCR assay of clinical samples. PGK1 was highly expressed in BC. **(G)** PCR assay revealed the expression of PGK1 in different cell lines. PGK1 was highly expressed in four BC cell lines compared to HBL-100 cell lines. **(H)** PGK1 was knocked down in MCF-7 and MDA-MB-231. **(I, J)** CCK-8 showed that the proliferation activity of the cells that knockdown PGK1 was dramatically reduced. *P<0.05, **P< 0.01, ***P<0.001.

A pan-cancer analysis of PGK1 expression levels showed that PGK1 was highly expressed in BC relative to normal tissues (**Figure 10A**). To further determine the prognosis of PGK1, survival analysis of overall survival (OS), disease-specific survival (DSS), and progression-free survival (PFS) was performed in TCGA, and patients with high PGK1 expression were shown to have a bad prognosis (*P*<0.05, **Figures 10B–D**). At the same time, we intensively analysed PGK1 relapse-free survival (RFS) in GSE22219 and obtained the same result (**Figure 10E**). Similarly, the GEPIA dataset showed similar results (**Supplementary Figure S2A**).

### 3.10 Experimental validation of PGK1

We further determined the function of PGK1 by *in vitro* experiments. We did the same validation with 20 pairs of breast cancer tissue samples from our hospital. In clinical samples, we observed similar expression trends (**Figure 10F**). As expected, PGK1 was highly expressed in 14 of the 20 pairs of tissue samples. **Figure 10G** shows that PGK1 is highly expressed in four BC cell lines compared to HBL-100 cell lines, and PGK1 expression was highest in MDA-MB-231 and MCF-7 cells compared to other breast cancer cells. These outcomes confirmed the accuracy of the bioinformatics studies mentioned above.

### 3.11 Experimental validation of PGK1

**Figure 11:**
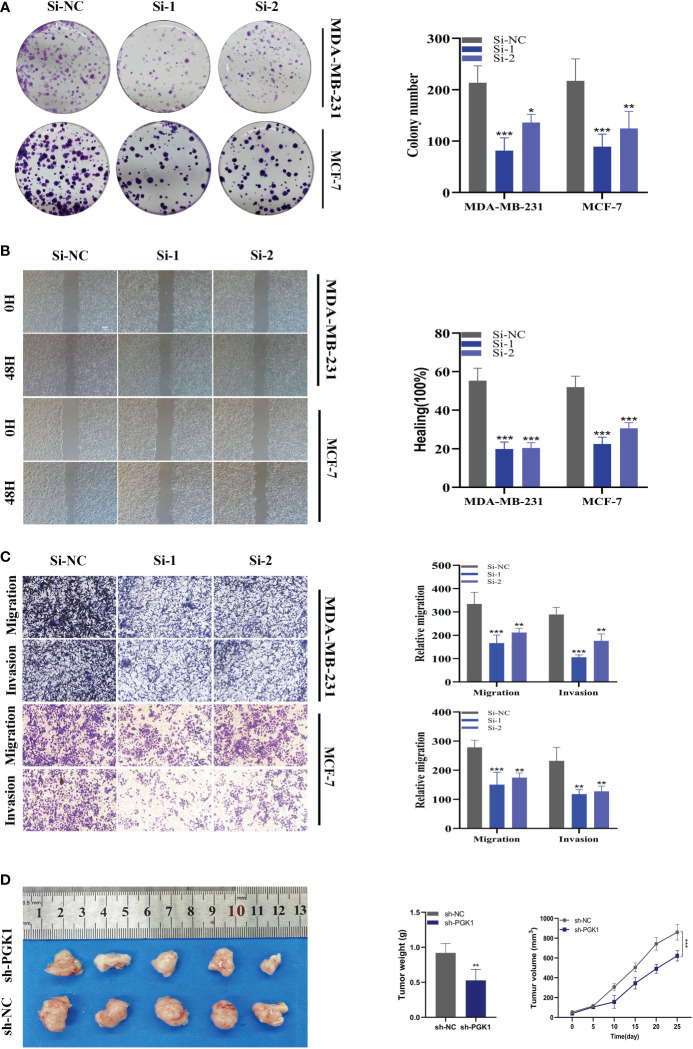
*In vitro* experiment after PGK1 knockdown. **(A)** After PGK1 knockdown, the cloning ability of MDA-MB-231 and MCF-7 cell lines decreased significantly. **(B)** Healing test. After PGK1 knockdown, the migration ability of MDA-MB-231 and MCF-7 cell lines decreased significantly. **(C)** Transwell assay. After PGK1 knockdown, the migration and invasion abilities of MDA-MB-231 and MCF-7 cell lines were significantly decreased. **(D)** Photographs of tumors obtained from the different groups of nude mice transfected with sh-NC, sh-PGK1. The average weight and tumor size were used to observe tumors. (*P<0.05, **P<0.01, ***P<0.001).

As mentioned previously, PGK1 was the highest expression level in MDA-MB-231 and MCF-7 cell lines, so we carried out gene knockdown in these two cell lines. These two cell lines saw a considerable change in PGK1 expression (**Figure 10H**). In CCK-8, we observed that the proliferation activity of PGK1 knockout MCF-7 cells was significantly reduced compared with the control cells (**Figure 10I**). Similar results were observed in cell line MDA-MB-231 (**Figure 10J**). To further verify the effect of PGK1 on the proliferation ability of BC cells, we also conducted cloning experiments. The results showed that the number and volume of colonies formed by the two cell lines decreased after the PGK1 gene knockdown (**Figure 11A**). Next, we conducted healing and transwell experiments to analyse the effects of PGK1 on the migration and invasion ability of BC cells. The results showed that the migration and invasion ability of BC cells were significantly weakened after PGK1 was knocked down (**Figures 11B, C**). As shown in **Figure 11D**, PGK1 knockdown inhibited tumor growth, resulting in decreased tumor volume and weight compared to control groups.

## 4 Discussion

The most frequent form of cancer in women, BC has a considerable negative impact on patients’ quality of life and comes at a significant financial cost to society (27). Existing conventional treatments have limited benefits for BC, and postoperative recurrence and drug resistance remain major issues in the clinical management of BC. The main causes of BC’s poor prognosis and treatment results are believed to be its high heterogeneity and complex TME (28).

By modulating signaling functions in the signaling network of tumor cells, sphingolipids and their metabolites contribute significantly to the maintenance of cell growth and signal transduction, controlling a variety of biological processes including growth, proliferation, migration, invasion, and metastasis (13). The functions of many sphingolipid regulators (mainly Cer, SPH, and S1P) are of great importance and are closely related to both the development and progression of cancer, so they can also be used in anticancer therapy. Eugen Ruckhäberle et al. date examined the expression of 43 genes related to the sphingolipid metabolic pathway in 1,269 BC samples using a gene microarray approach. Investigators found that sphingosine kinase 1 (SPHK1), ceramide galactosyltransferase (UGT8), and ganglioside GD3-synthase (ST8SIA1) showed high expression activity in estrogen receptor-negative BC. In contrast, glucosylceramide synthase (GCS), dihydroceramide synthase (LASS4, LASS6), and acidic ceramidase (ASAH1) were expressed at higher levels in estrogen receptor-positive BC (29). The study also found that SPHK1 expression correlated with prognosis, with 75. 8 ± 1. 9% of patients with low SPHK1 expression being metastasis-free at 5 years, while only 64. 9 ± 3. 6% of patients with high SPHK1 expression were metastasis-free at 5 years. As more research has been conducted, more findings have shown that SPHK1 protein levels in BC patients correlate with the grade of tumor progression and are potential indicators of tumor malignancy grading (30, 31).

This study explored the role of SRGs in BC by combining single-cell sequencing and WGCNA. Seven SRGs were screened by Cox regression and Lasso regression analysis, and prognostic models of BC were created using these seven genes. The model divides patients into high and low-risk groups based on median risk values by assessing each patient’s risk score, with the high-risk group having a significantly lower prognosis than the low-risk group. To validate the accuracy of the model, we performed ROC curves on both the training cohort and the test cohort, with AUC values greater than 0.7 at 1, 3, and 5 years, while the maximum AUC value (0.837) was detected at 5 years. In addition, clinically relevant ROC curves and decision curves show that risk scores outperform other clinical characteristics in terms of the efficiency of clinical application. The proportion of stage II-IV patients was greater in the high-risk group compared to the low-risk group, which is consistent with the traditional clinical grading. These findings imply that the model can more accurately forecast a BC patient’s prognosis. We also identified four chemotherapeutic agents that were sensitive in the high-risk group, namely CP724714, Lapatinib, WZ3105, and Pyrimethamine. Among them, Lapatinib has been approved for use in patients with advanced or metastatic BC (32, 33); CP724714 (HER2/ErbB2 inhibitor) is also in clinical trials for BC patients (34–36); and the FDA-approved antibacterial drug etanercept (PYR) has shown therapeutic activity in mouse models of BC, with direct tumor suppression and immunostimulatory effects (37). These outcomes emphasise how accurate our approach is at predicting outcomes and treating BC patients.

In the growth of tumors, the TME is crucial. The prognosis of patients may be impacted by the different immune cells’ tumor invasion (38, 39). Previous studies have reported that M1 macrophages in BC are associated with increased tumor cell apoptosis and decreased metastasis, while M2 types are associated with invasion and metastasis (40, 41). In BC TME, Tumor-associated macrophages (TAMs) are often present as the M2 type and play a role in promoting BC progression (42). Our study found more M1 macrophage infiltration in the low-risk group and more M2 macrophages in the high-risk group, which also corroborates previous work. In our study, TMB levels were also found to be positively correlated with risk scores. Previous studies have shown that patients with higher TMB levels may show better sensitivity to immunotherapy (43). We further performed a survival analysis and found that the high-risk group with a high tumor mutation burden, had the worst prognosis, suggesting that patients in the high-risk group may show better sensitivity to immunotherapy. Based on this, we compared the TIDE scores of the two patient groups and discovered that the high-risk group’s TIDE ratings were lower, which again raises the possibility that these individuals may be more responsive to immunotherapy. These results offer more evidence that our risk model is connected to the immunological environment and can forecast the outcome of BC patients.

The gene with the greatest HR in our created signature is PGK1 (phosphoglycerate kinase 1), which has been linked to a bad prognosis in BC. Our cellular assays show that PGK1 is highly expressed in breast cancer and that knockdown of PGK1 expression greatly reduces the activity, invasion, and migration ability of BC cells. This adds to the evidence that PGK1 plays a role in BC. Many previous studies have shown that PGK1 has a function in malignant tumors. Therapeutic inhibition of PPARα-HIF1α-PGK1 signaling targeting acute myeloid leukemia according to Jie Zha et al (44). Overexpression of PGK1 in prostate cancer cells has been reported to increase cell metastasis through the CXCR4/CXCL12 axis (45). PGK1-mediated phosphorylation of Beclin1 at Ser30 is positively associated with poor prognosis in glioblastoma (46). In our study, PGK1 was also found to be a potential target for BC.

## 5 Conclusion

In conclusion, our results suggest that the model constructed with 7 SRGs can predict the prognosis of BC patients well. Furthermore, we have verified the function of PGK1 in BC through cellular experiments and screened candidate vaccine genes for BC. These results might offer useful information for creating fresh BC treatment plans.

## Data availability statement

The original contributions presented in the study are included in the article/**Supplementary Material**. Further inquiries can be directed to the corresponding authors.

## Ethics statement

The studies involving human participants were reviewed and approved by The First Affiliated Hospital of Nanjing Medical University’s Institutional Ethical Board gave the study its approval. The patients/participants provided their written informed consent to participate in this study. The animal study was reviewed and approved by The Committee on the Ethics of Animal Experiments at Nanjing Medical University.

## Author contributions

HX and YX participated in the design of the study. SP and PZ provided the idea for the project and participated in the design of the experimental scheme. LY, YK, and YD performed the experiments. HC, MZ, and SZ contributed to data collection and analysis. HX and YX confirm the authenticity of all the raw data. All authors contributed to the article and approved the submitted version.
