# Supplementary material for: Exploring the role of sphingolipid-related genes in clinical outcomes of breast cancer
Source: Front Immunol. 2023 Feb 13;14:1116839. doi: 10.3389/fimmu.2023.1116839 (PMC9968761; doi:10.3389/fimmu.2023.1116839)
Supplement: Supplementary file 2 [file Table_2.docx]

| **Oligonucleotides** | **Nucleotide sequence (5'-3')** |
| --- | --- |
| **siRNA** |  |
| Scramble control | GCUUCGCGCCGUAGUCUUA |
| Si-PGK1-1 | GAGTCAATCTGCCACAGAA |
| Si-PGK1-2 | CCAAGTCGGTAGTCCTTAT |
|  |  |
| **Primer** |  |
| GAPDH | GGCCTCCAAGGAGTAAGACC (forward) |
|  | AGGGGAGATTCAGTGTGGTG (reverse) |
| PGK1 | TCACTCGGGCTAAGCAGATT (forward) |
|  | CAGTGCTCACATGGCTGACT (reverse) |
|  |  |

**Table S2. Oligonucleotides used in research**
