# Supplementary material for: Exploring the role of sphingolipid-related genes in clinical outcomes of breast cancer
Source: Front Immunol. 2023 Feb 13;14:1116839. doi: 10.3389/fimmu.2023.1116839 (PMC9968761; doi:10.3389/fimmu.2023.1116839)
Supplement: Supplementary file 3 [file DataSheet_1.docx]

Supplementary Material

# Supplementary Figures


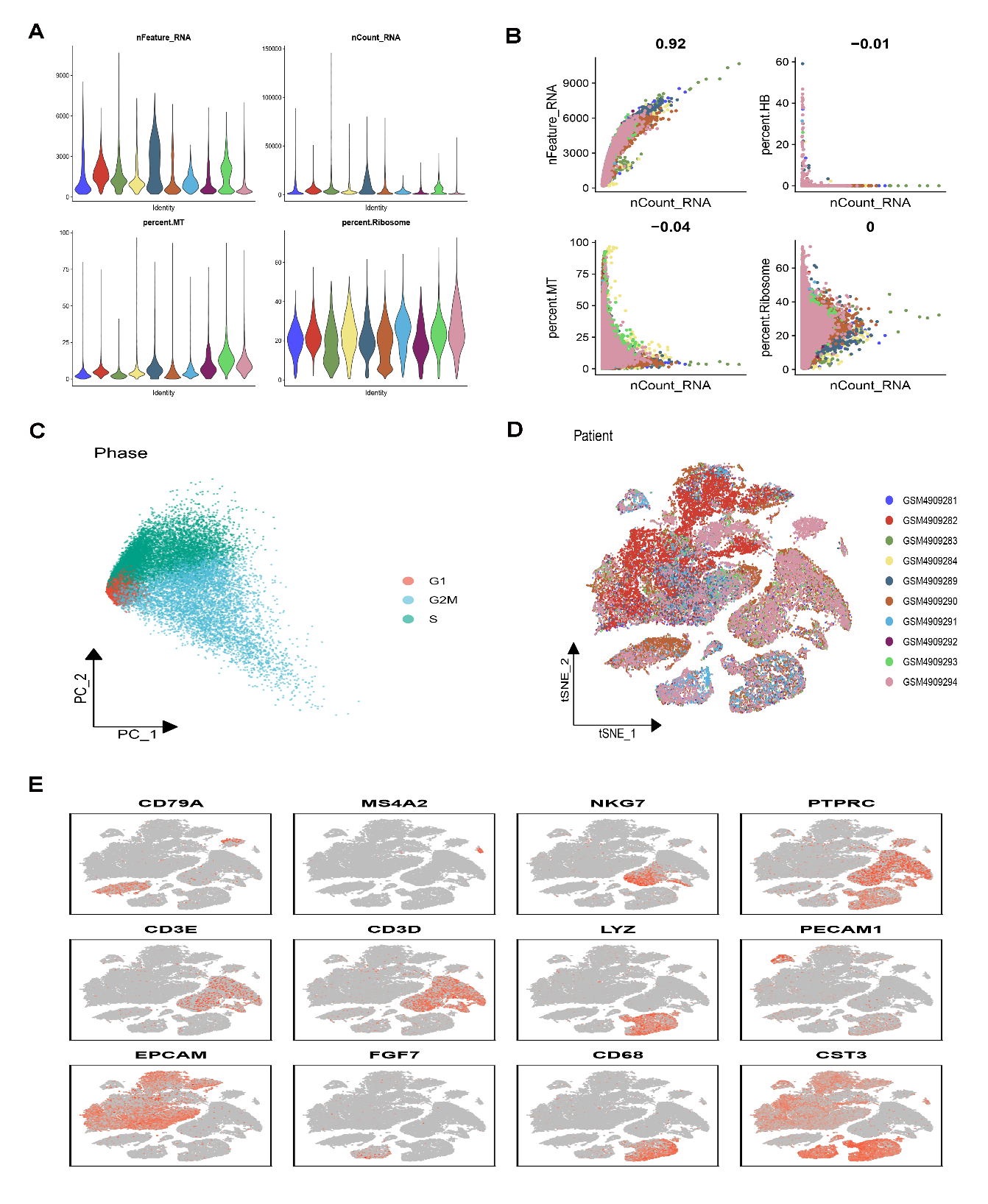


**Supplementary Figure S1.** Quality control and dimension reduction analysis of single-cell sequencing data. (A)By limiting the ratio of mitochondrial, ribosomal, and erythrocyte genes, cells that did not meet the criteria were removed. (B)Sequencing depth and total intracellular sequences exhibit significantly substantial positive associations (R=0.92). (C)The principal component analysis explored whether the cell cycle had any influence on the analysis. (D)The cell distribution of the samples showed no significant batch effect. (E)The expression of the genes that identify the cell type.


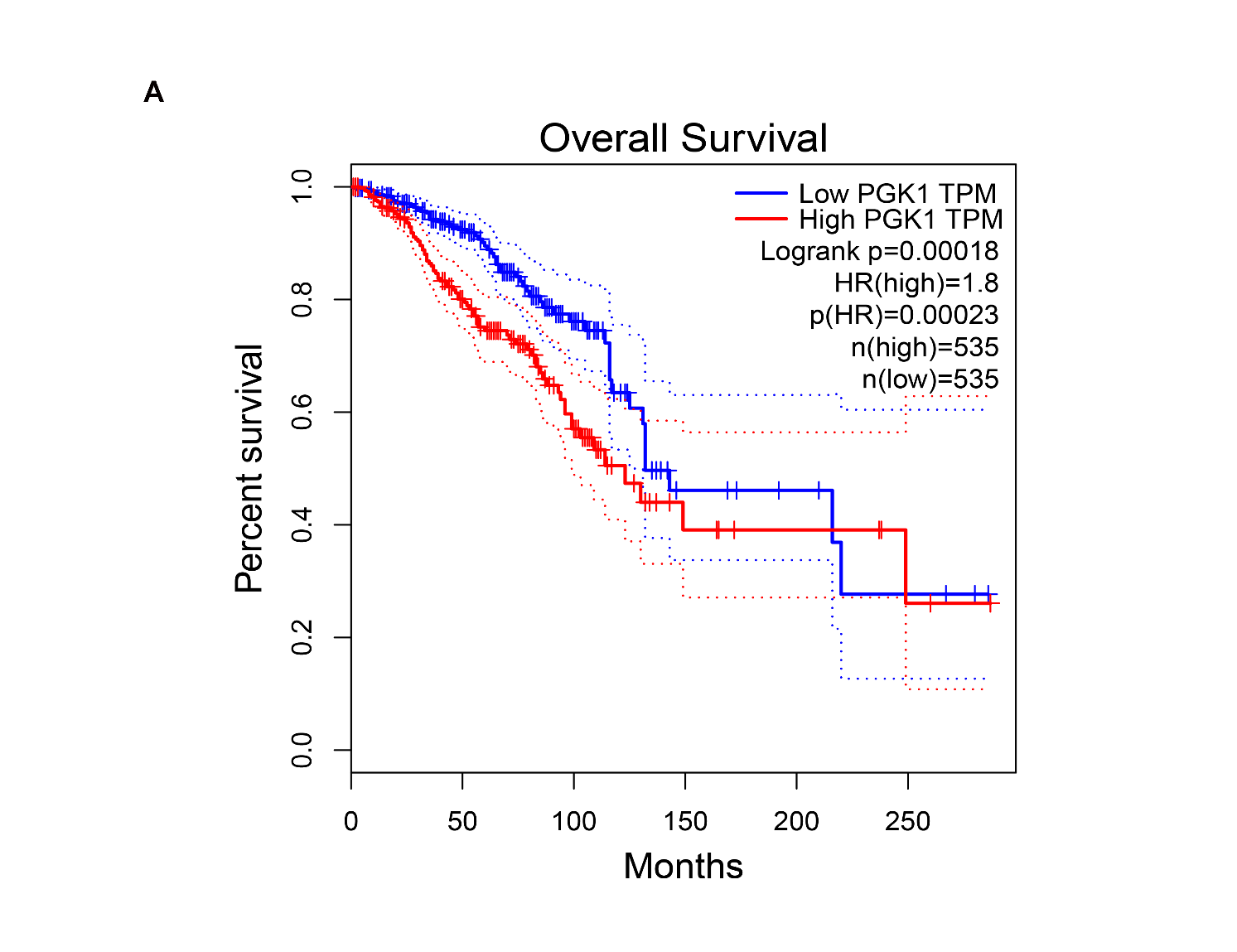


**Supplementary Figure S2.** A survival curve plot. (A) GEPIA dataset was used to verify the effect of PGK1 on the prognosis of LUAD patients.
